# Supplementary figures and images for: Incidence and Outcome of Patients with Cardiogenic Shock and Detection of Herpes Simplex Virus in the Lower Respiratory Tract
Source: J Clin Med. 2022 Apr 22;11(9):2351. doi: 10.3390/jcm11092351 (PMC9105969; doi:10.3390/jcm11092351)

Figure S1: Patient selection

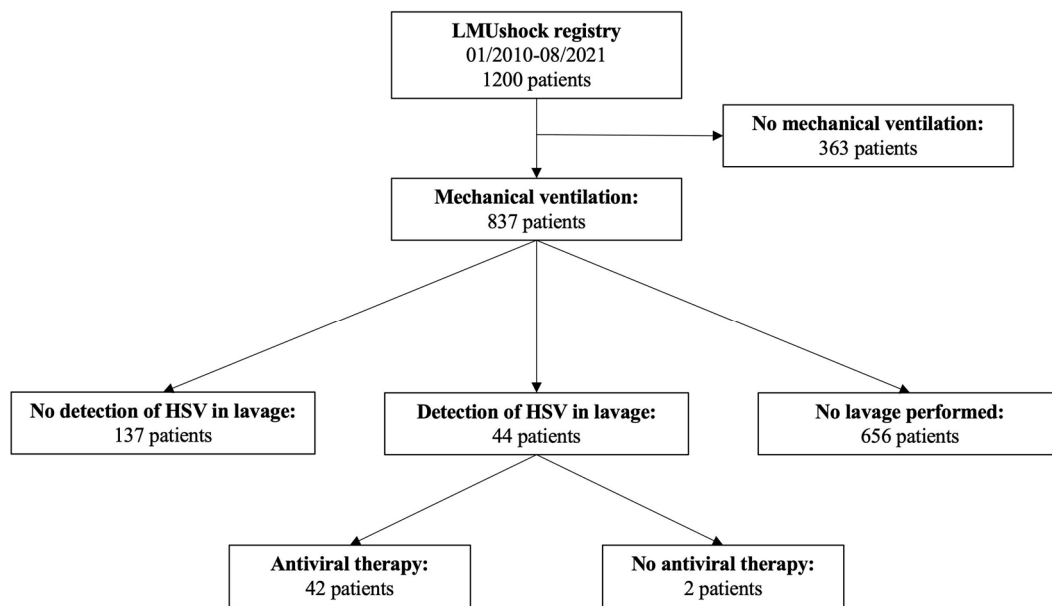

Supplement: Supplementary file 1 [file jcm-11-02351-s001.zip › jcm-1646001-supplementary.pdf]
